# Risk of Fire and Explosion in Electrical Substations Due to the Formation of Flammable Mixtures

**DOI:** 10.1038/s41598-020-63354-4

**Published:** 2020-04-14

**Authors:** Mohanad El-Harbawi, Fahad Al-Mubaddel

**Affiliations:** 1 Department of Chemical Engineering, King Saud University, Riyadh, 11421 Saudi Arabia; 2 Fellow, King Abdullah City for Renewable and Atomic Energy: Energy Research and Innovation Center, (ERIC), Riyadh, 11451 Saudi Arabia

**Keywords:** Chemical engineering, Fossil fuels, Environmental sciences, Risk factors, Engineering

## Abstract

Transformers reduce the voltage from overhead powerlines to voltages acceptable for city/neighbourhood needs. Overheating of transformer cooling fluids presents a serious hazard. In this work, the risk of fires and explosions due to vaporisation of the hydrocarbon components of mineral oil, which is used as a transformer cooling fluid in electrical substations, was investigated. The compositions of new and used mineral oil from an electrical substation in Riyadh were analysed using gas chromatography–mass spectrometry, and C_6_ to C_41_ hydrocarbons were detected. The majority of the components were alkanes, alkenes, or alkynes; some ketone, alcohol, aromatic, and anhydride species were also detected. Approximately 25% of the compounds comprising the new oil sample were alkanes, whereas more than 33% of the used oil sample components were alkanes. The lower and upper flammability limits (LFL and UFL) of the mixtures were found to be 0.88 and 5.75 vol.% for the new oil and 0.47 and 3.05 vol.% for the used oil, respectively. These values were used to construct a flammability diagram. The results indicated that the new and used oil vapour mixtures were not flammable at 25 °C and 1 atm, but would become flammable at 77 and 115 °C.

## Introduction

Electrical energy is a necessity for all aspects of civilisation. Increasing electrical energy consumption and demand have made the world highly reliant on electrical power systems. It is expected that most of the world’s power will come from solar sources by 2050^1,2^. Solar cell technology has recently attracted significant attention due to the excellent photovoltaic performance of current solar cell technology^3–5^. However, in many countries, electricity is currently produced by fossil fuel combustion in thermal power plants^6^. As these plants are typically located outside citylimits, the electrical energy needed for households and other activities is transported through powerlines to substations or transformers. Transformers are electrical devices used to convert an alternating current (AC) at a certain voltage to another AC voltage using the principles of electromagnetism and electromagnetic induction^7^. The voltage conversion process generates heat due to electrical resistance; insulating fluids are used to absorb this heat.

Three types of transformers are commonly used: (i) less-flammable-liquid-filled transformers, (ii) flammable-liquid-filled transformers, and (iii) non-liquid (dry type) transformers^8^. Dry-type transformers (sometimes called cast resin transformers) use gases or dry compounds as the insulation material, and are generally considered safer than liquid-type transformers^8^. However, they are costly and bulky.

The liquid inside transformers is referred to as the insulant, insulating liquid, or dielectric liquid^9^. Petroleum-based mineral oils have been used as transformer insulating liquids since 1887^10^, and most modern electrical power transformers use mineral oils derived from petroleum crude oil for cooling and insulation. These oils comprise various classes of hydrocarbon compounds, including naphthalenes, paraffins, iso-paraffins, and aromatics^11,12^. According to the United States Environmental Protection Agency, the main component of most commercially available mineral-oil-based transformer fluids is hydrotreated light naphthenic petroleum distillate^13^. These oils can leak due to gasketing, cracked insulation, or loose manhole covers^14^, resulting in environmental problems due to their toxicity^11,15,16^ and even fire and/or explosion accidents via direct contact of the leaked oil with high-voltage elements^17,18^. When the internal temperature of a transformer reaches 150–300 °C under abnormal conditions, the mineral oils produce the gases hydrogen and methane via chemical decomposition. At temperatures above 300 °C, ethylene is formed, and above 700 °C, large amounts of hydrogen and ethylene are produced^19^. These gases tend to dissolve partially or entirely in the mineral oil^20^ and can form combustible/flammable mixtures if they escape the transformer oil compartment, leading to unexpected fire/explosion accidents.

Mineral oil is a highly refined hydrocarbon-based oil and has been the most widely used insulation liquid in electrical applications for more than 100 years^21^. The most important fire safety parameters of fluids are their fire point and flash point^22^. A fluid will only ignite if it its temperature reaches its fire point and it is exposed to an ignition source. As a fluid is heated, the concentration of vapour above the fluid increases. At the flash point, the resulting vapour-air mixture can be ignited; when the temperature is further increased to the fire point, combustion will be sustained on the fluid surface. The fire point and flash point of mineral oil are ~165 and ~145 °C, respectively^23,24^. That is, under typical ambient conditions mineral oil may burn, but will it not ignite readily^23^. However, due to excessive heating, mineral oil may become combustible or flammable. It has been reported that 70% to 80% of all transformer failures are due to internal winding insulation failure^25^. This type of fault may lead to fire and explosion due to the decomposition and vaporisation of the oil and the subsequent formation of gas bubbles^8^. The exact conditions under which mineral oil will become flammable depend on its composition. The composition of transformer oil can be identified using various analytical methods, such as gas chromatography–mass spectrometry (GC–MS) and gas chromatography–isotope ratio mass spectrometry (GC–IRMS). GC analysis is sufficient to identify the compounds present in a sample and the percentage of each one^26^.

A vapour/air mixture will only ignite and burn when its concentration lies between the lower flammability limit (LFL) and upper flammability limit (UFL)^27^. Outside this range, the oxygen or fuel concentration will be insufficient to sustain combustion. These flammability limits can be measured using various apparatuses or determined using empirical equations^28^. Several methods and databases provide flammability limit information for hydrocarbons^29,30^. The most popular flammability limit databases are the one published by the American Institute of Chemical Engineers (DIPPR 801)^31^ and the extensive database provided by Yaws^32^.

Transformer fires and explosions can occur for a variety of reasons. The most common is a lightning strike, which can damage wires and/or equipment and cause too much electricity to flow into the transformer, leading to fire and/or explosion^33^. Strong rain and high winds can cause trees to fall on transformers, causing explosions. Although transformer fires and explosions are highly unlikely under normal weather conditions, they can potentially occur due to design faults, faulty hardware, or an overload in the system. Sudden damage to transformers can also lead to overcharging, which can create sufficient quantities of heat and sparks to ignite the mineral oil. The gases generated by boiling mineral oil create a massive overpressure inside the sealed transformer, leading to oil tank rupture, which results in the release of large amounts of energy and strong thermal radiation, scattering flaming oil, gaseous decomposition products, solid insulation, and molten conductor material over the surrounding area^17,34^. The explosion and thermal radiation generated by transformer failure have been reported to ignite neighbouring transformers more than 18 m from the initial fire. The temperature of oil fires ranges from 960 to 1200 °C, and a transformer oil fire can last from 4 to 28 h^34^. However, transformer tank explosions do not always result in a fire; the possibility of fire depends on how quickly the transformer protection system operates^35^. However, even when no fire occurs, the release of oil onto the site can cause major environmental pollution.

Transformer fires are generally of two types: pool fires and spray fires. A pool fire can occur when transformer oil leaks onto the ground via gaskets, holes in the radiator fins or the steel tank, or similar defects. Over time, the oil accumulates on the ground, forming an oil pool that can easily burn if ignited, resulting in a pool fire. Spray fires occur when the mineral oil inside the storage container is heated due to internal insulation failure. The temperature of the mineral oil inside the transformer tank increases, forming flammable vapour that is suddenly released from an opening in the tank into the surrounding atmosphere.

Although the probability of transformer fires and explosions is relatively low, it is not negligible. Transformer fires are difficult to extinguish and control. Additionally, mineral oil fires can spread to nearby equipment and buildings, presenting a high risk^36^. Several notable transformer fire accidents have occurred in the past. Perhaps the worst transformer accident occurred in a coalmine in western Turkey in May 2014 when an electrical fault resulted in a transformer explosion and a fire. More than 200 people were killed in the disaster, and 80 were injured^37^.

To the best of our knowledge, most previous studies related transformer explosions and fires have focused on investigation of the electrical and mechanical failure of the transformers. In contrast, this workis aimed at investigating the possibility of fire and explosion due to the formation and release of flammable mixtures from power transformers.

## Materials and Methods

### Sample collection

Two samples of mineral oil (new and used) were collected from an electric power substation in Riyadh. The new oil was still in its original container and had never been used. The used oil had been filled into a transformer tank, and the transformer was operated for a maximum period of one year. In electrical stations, transformer oil is usually replaced with new oil after one year regardless of whether the transformer has been operated. These samples were stored in 1 L bottles, which were tightly closed and stored in a safe place in a laboratory cabinet under normal conditions.

### Compositional analyses

GC-MS analysis was conducted using a procedure based on our previous study^38^. The two oil samples were diluted with *n*-hexane before GC-MS analysis (Shimadzu GCMS-QP20 Ultra). The following GC-MS settings were used: electron impact ionisation, electron energy, 70 eV, scan range: 50 to 550 amu at scan rate of 1 scan per second. Helium (purity 99.999%) was used as the carrier gas at a fixed flow rate of 50 mL/min, with a linear velocity of 47.4 cm/s and a column inlet pressure 100 kPa. The end of the column was connected to the ion source of a mass-selective detector operated in electron impact ionisation mode. The samples were injected into a HP5-fused silica (5% phenyl polysilphenylene-siloxane) capillary column (CPWAX 58-FFAP; length: 50 mm; diameter: 0.32 mm; film thickness: 0.20 mm). The oven temperature ramp rate was fixed at 4 °C/min; the initial temperature of 50 °C was held for 2 min, after which it was increased to 220 °C over 30 minutes and then held at this temperature for 30 min. The components were analysed and identified via computer spectral matching methods by matching their mass spectra with data obtained from the National Institute of Standards and Technology (NIST) database.

The mass fraction of each compound in the liquid phase was calculated using the ratio of the area of the peak corresponding to that compound to the total area of all compounds (Eq. 1):

1 $${X}_{i}=\frac{{A}_{i}}{{A}_{T}}$$

where

*X*_*i*_ represents the mass fraction of component *i* (%),

*A*_*i*_ represents the peak area of component *i*, and

*A*_*t*_ represents the peak area of all components.

The mass fraction was then converted into the corresponding mole fraction as follows:

2 $${x}_{i}=\frac{{X}_{i}/{M}_{i}}{\sum {X}_{i}/{M}_{i}}$$

where

*x*_*i*_ represents the mole fraction of component *i* in the liquid phase, and

*M*_*i*_ represents the molar mass of component *i*.

### Composition of the vapour phase

Vaporisation characteristics are important to investigations of flammability. Some components of mineral oil vaporise at ambient temperature or can produce flammable mixtures if exposed to heat. Therefore, the composition of the vapour phase must be identified. The amount of liquid vaporised was estimated assuming that Raoult’s Law was applicable (Eq. 3). According to Raoult’s law, the vapour phase composition can be calculated:

3 $${x}_{i}{P}_{i}^{sat}={y}_{i}{P}_{t},$$

where

*x*_*i*_ represents the mole fraction in the liquid phase of component *i*,

${P}_{i}^{sat}$ represents the vapour pressure of compound *i*,

*y*_*i*_ represents the mole fraction of component *i* in the vapour phase (%), and

*P*_*t*_ represents the total pressure.

The vapour pressure of each component at 25 °C and 760 mmHg was taken from the ChemSpider website (www.chemspider.com).

### Determination of LFL and UFL

In the absence of experimental data, flammability limits can be predicted using established theoretical methods. Jones^39^ found that in the formation of hydrocarbon vapours, the flammability limits were functions of the stoichiometric concentration of the fuel, *C*_*st*_ (Eqs. 4 and 5):

4 $$LFL\,=\,0.55\,{C}_{st}$$

5 $$UFL\,=\,3.5\,{C}_{st}$$

where

0.55 and 3.5 are constants, and

*C*_*st*_ represents the volume percent of the fuel in the fuel + air mixture (expressed by Eq. 8).

For most organic compounds, the stoichiometric concentration can be determined using the following general combustion reaction:

6 $${{C}}_{{m}}{{H}}_{{x}}{{O}}_{{y}}+{z}{{O}}_{{2}}\to {mC}{{O}}_{{2}}+\left(\frac{{x}}{{2}}\right){{H}}_{{2}}{O}$$

where *z* represents the equivalent moles of O_2_ divided by the moles of the fuel and can be expressed as

7 $${z}={m}+({x}/{4})-({y}{/}{2})$$

The stoichiometric concentration, *C*_*st*_, can be determined as a function of *z*:

8 $$=\,\frac{{100}}{\left[{1}+\left(\frac{{z}}{{0.21}}\right)\right]}$$

The LFL and UFL can be determined by substituting Eq. 7 into Eq. 8 and applying Eqs. 4 and 5:

9 $${LFL}=\frac{{0.55}({100})}{{4}{.76m}+{1}{.19x}-{2}{.38y}+{1}}$$

10 $${UFL}=\frac{{3.50}({100})}{{4}{.76m}+{1}{.19x}-{2}{.38y}+{1}}$$

The LFL and UFL values of mixtures can be calculated according to the Le Chatelier equations^40^ (Eqs. 11 and 12).

11 $${LF}{{L}}_{{mix}}=\frac{{1}}{\sum ({y}_{{i}}{/}{LF}{{L}}_{{i}})}$$

12 $${UF}{{L}}_{{mix}}=\frac{{1}}{\sum ({{y}}_{{i}}{/}{UF}{{L}}_{{i}})}$$

Here,

${LF}{{L}}_{{i}}$ represents the LFL of component *i* (in vol.%) in the fuel and air mixture,

${UF}{{L}}_{{i}}$ represents the UFL of component *i* (in vol.%) in the fuel and air mixture, and

*n* represents the number of combustible species.

Zabetakis *et al*.^41^ reported that the LFL decreases and the UFL increases with increasing temperature. This means that an increase in temperature widens the flammability range. The following empirical equations were derived for vapours:

13 $${LFL}{(}{T}{)}={LFL}{(}{298K}{)}-\frac{{0.75}}{{\Delta }{{H}}_{{c}}}({T}-{298})$$

14 $${UFL}{(}{T}{)}={UFL}{(}{298K}{)}+\frac{{0.75}}{{\Delta }{{H}}_{{c}}}({T}-{298})$$

where

*∆H*_*c*_ represents the net heat of combustion (kcal/mol),

*T* represents the temperature (in K), and

LFL and UFL are given in vol.%.

### Determination of limiting oxygen concentration

The limiting oxygen concentration (LOC), which is also called the minimum oxygen concentration, is defined as thelowest concentration of oxygen in a fuel-air-inert gas mixture needed to propagate a flame^27,42^. The LOC can be estimated using the following simple method^43^:

15 $${LOC}=\left(\frac{{moles}\,{of}\,{fuel}}{{total}\,{moles}}\right)\left(\frac{{moles}\,{of}\,{{O}}_{{2}}}{{moles}\,{fuel}}\right)={LFL}\left(\frac{{moles}\,{of}\,{{O}}_{{2}}}{{moles}\,{fuel}}\right)=z(LFL)$$

For mixtures, the LOC can be estimated using Eq. 16^42^:

16 $${LO}{{C}}_{{mix}}=\sum {y}_{i}\,{z}_{i}/\sum {y}_{i}/{L}_{i}^{\ast }=\sum {y}_{i}\,{z}_{i}/\sum {y}_{i}\,{z}_{i}/LO{C}_{i}$$

17 $${L}_{i}^{\ast }=LO{C}_{i}/{z}_{i}$$

where

*LOC*_*mix*_ represents the LOC of the vapour mixture (vol.%),

*z* represents the equivalent moles of O_2_ divided by the moles of fuel for compound *i* in the vapour phase, and

*LOC*_*i*_ represents the LOC for an individual compound (Eq. 15).

## Results and Discussion

### Components of the oil samples and their mass and mole fractions

#### Components

**Figure 1:**
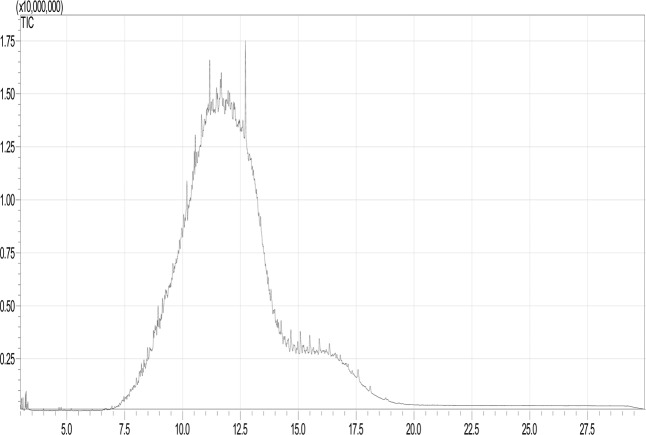
GC-MS chromatogram of the new oil sample.

**Figure 2:**
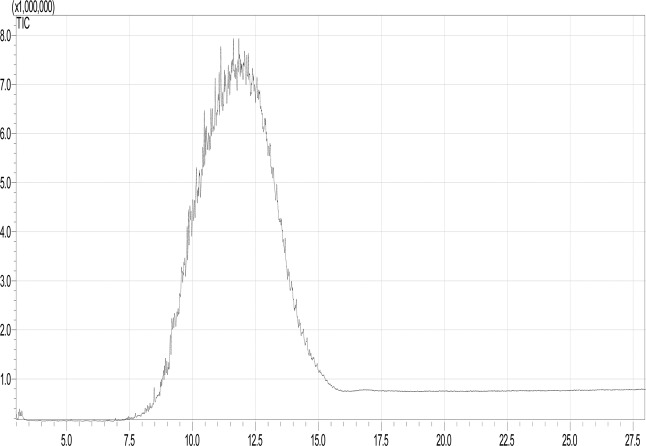
GC-MS chromatogram of the used oil sample.

The two samples of oil were analysed using GC–MS. Their components were identified based on the retention time, component formula, molecular mass, boiling point, match percent, and NIST library number. Figure 1 shows the GC-MS mass chromatogram for the products detected in the liquid phase for the new oil. Table S1 presents the details of these components. This table also contains some properties that were used to calculate the LFL, UFL, and the LOC for the mixture. The results indicated that the new oil sample contained 33 hydrocarbon components, C_8_ to C_35_. The majority of these components were alkanes, alkenes, and alkynes, along with some ketone, alcohol, aromatic, and anhydride species. Peaks 24, 25, 26, and 29 constituted approximately 25% of the sample. These peaks corresponded to alkanes, namely, octadecane, 2,6,10,14-tetramethylpentadecane, 2,6,10,14-tetramethylhexadecane, and dotriacontane. The components of the used oil sample were identified via the same procedure; the corresponding GC-MS mass chromatogram is shown in Fig. 2. Table S2 presents the details of these components. The used oil sample contained27 hydrocarbon components ranging from C_6_ to C_41_. The majority of the components were alkanes and alkenes, with some ketone, aldehyde, alcohol, ether, and ester species. However, more than 33% of the components were alkanes.

### LFL, UFL, and LOC of each component and the mixtures

The LFL and UFL for each component in the two samples were obtained from the well-known database DIPPR Project 801^31^. Some components were not available in this database or in other published literature. Hence, the stoichiometric concentration method (Eqs. 9 and 10) was used to calculate the unavailable values. The LFL and UFL values are presented in Tables S1 and S2.

The LFL and UFL of the mixtures (LFL_mix_ and UFL_mix_, respectively) were computed using the Le Chatelier equations (Eqs. 11 and 12). For the new oil sample, the values of LFL_mix_ and UFL_mix_ were calculated to be 0.88 and 5.75, respectively; those of the used oil sample were 0.47 and 3.05. The LOCs for each component and for the mixture were calculated using Eqs. 15 and 17, respectively. For the new and used oil samples, the calculated LOC_mix_ values were 11.26 and 11.38 vol.%, respectively.

### Construction of the flammability diagram

Flammability diagrams play an important role in elucidating the flammability of substances and mixtures and preventing fire and explosion accidents. They are frequently used in industry to determine whether flammable mixtures will be formed during industrial processes^28^. Flammability diagrams depict the flammability region for mixtures of fuel, oxygen, and an inert gas (such as N_2_, CO_2_, Ar, He, etc.). To date, very few flammability diagrams have been experimentally determined. To the best of our knowledge, no flammability diagrams have been measured for mineral oil–oxygen–nitrogen mixtures.

**Figure 3:**
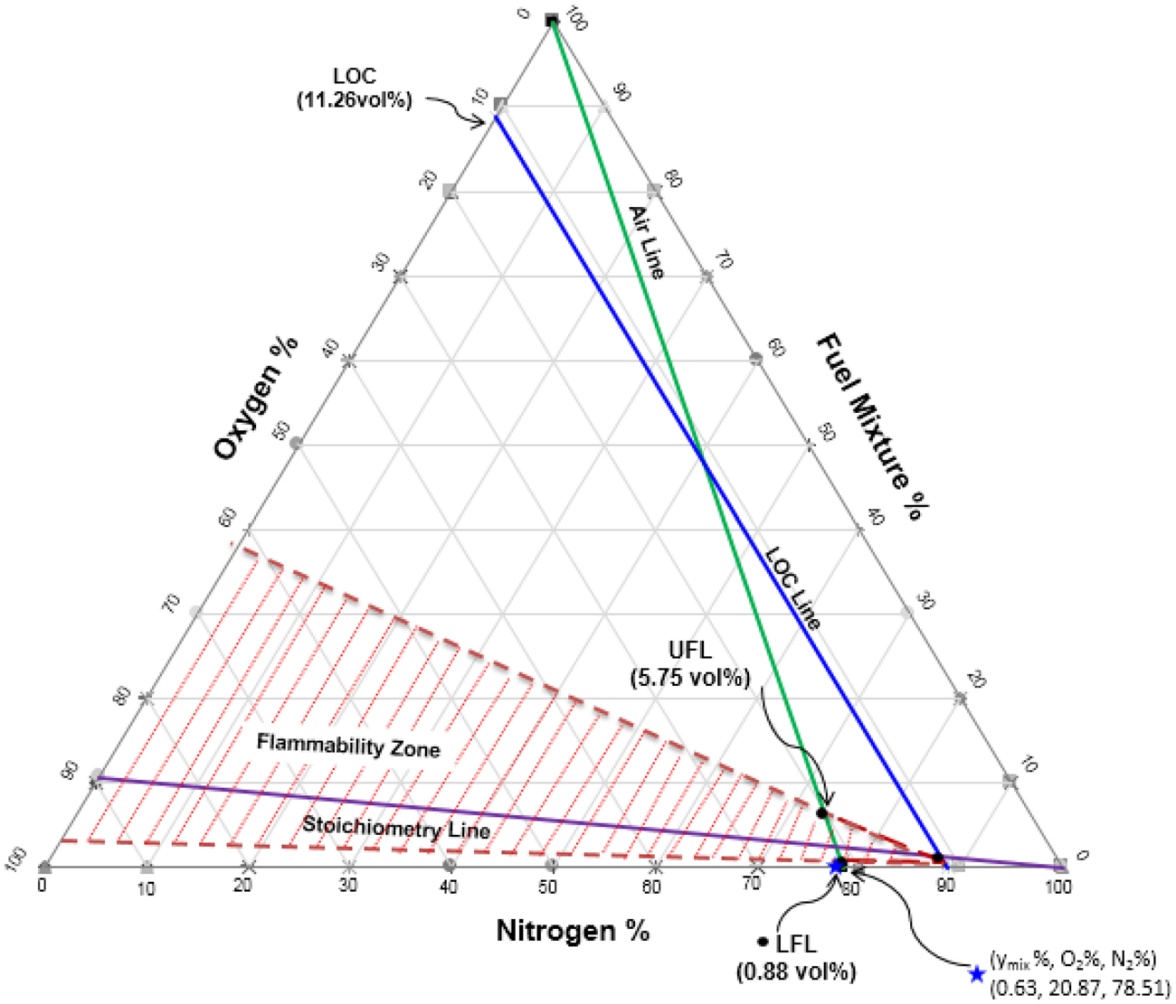
Triangular flammability diagram for the new oil–vapour mixture at 25 °C.

**Figure 4:**
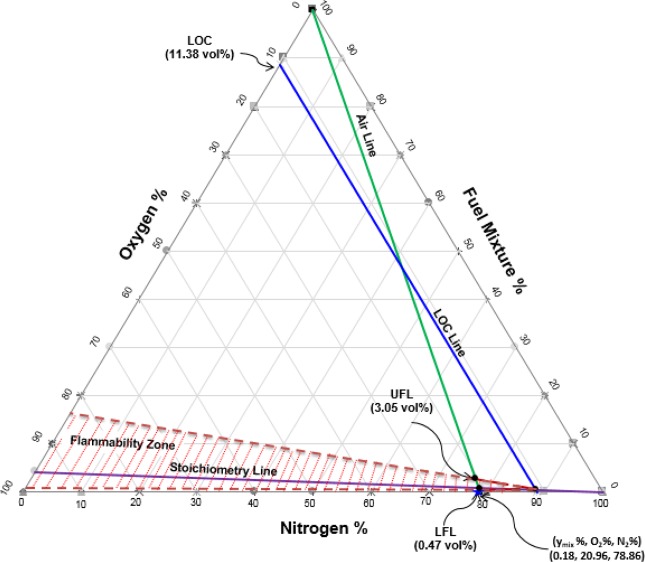
Triangular flammability diagram for the used oil–vapour mixture at 25 °C.

To construct the flammability diagram for the vapour mixtures of the two mineral oil samples, the percentages of fuel, O_2_, and N_2_ (in vol.% or mol.%) were required, along with the LFL, UFL, and LOC values of the mixture. The airline was plotted using the air compositions in Tables S1 and S2 (for the new oil, 78.51% nitrogen and 20.87% oxygen; for the used oil, 78.86% nitrogen and 20.96% oxygen). The stoichiometric line was drawn by locating the stoichiometric point (calculated using Eq. 8) on the oxygen axis, and then drawing a line from this point to the 100% point of the nitrogen axis^28^. The LOC_mix_ line was drawn by locating the LOC_mix_ values (11.26% and 11.38% for the new and used oil, respectively) on the air axis and then drawing a line parallel to the fuel axis until it intersected the stoichiometric line. This identified the nose of the flammability diagram. To identify the flammability zone, the values of LFL_mix_ and UFL_mix_ were located on the airline; the flammability zone is the area to the right of the airline. Figures 3 and 4 depict the triangular flammability diagrams for the vapour mixtures of the new oil and used oil, respectively. The y_mix_, N_2_, and O_2_ values of the two vapour mixtures were located slightly outside the flammable zones. Therefore, the vapour mixtures were concluded not to be flammable at 25 °C and 1 atm. However, the compositions of these mixtures were located close to the flammable zone boundaries, indicating that the mixtures could become flammable if the temperature were increased sufficiently.

To prevent a flammable mixture from forming, the temperature at which the vapour mixture would become flammable was predicted. First, the LFL and UFL were calculated at a temperature other than 25 °C using Eqs. 13 and 14, respectively. These equations require the heat of combustion for each component in the two samples. For most of the components, this value could be obtained from various resources^32,44–46^. Values that were not available from these references were obtained using Hess’s law of constant heat summation. The second step was to calculate the vapour pressure for each component at the new temperature.

**Figure 5:**
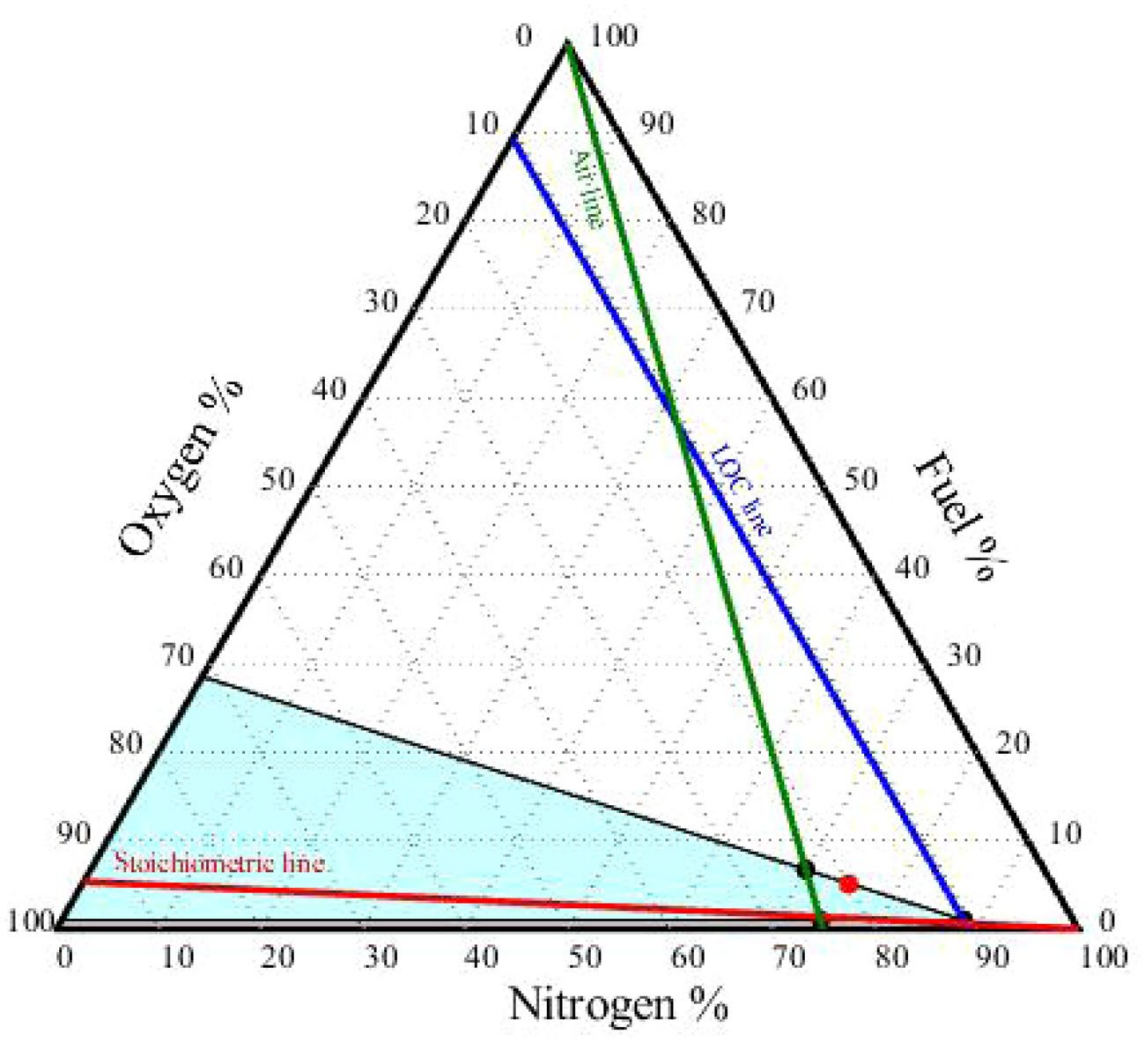
Triangular flammability diagram of the new oil vapour mixture at 70 °C.

**Figure 6:**
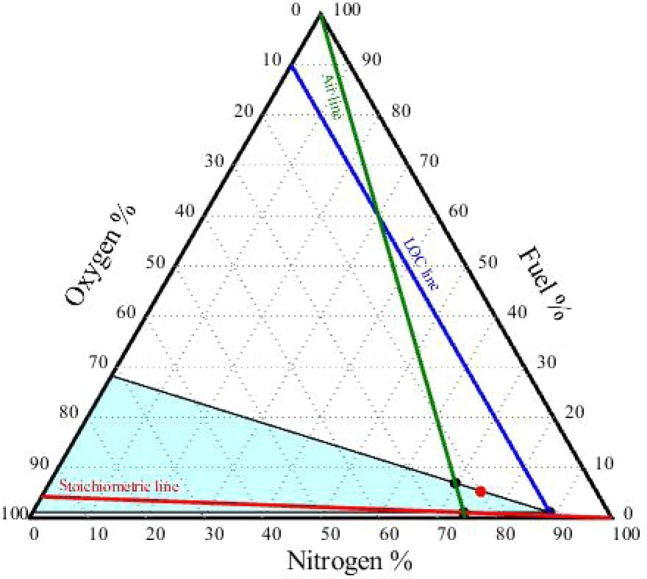
Triangular flammability diagram of the used oil vapour mixture at 115 °C.

The vapour pressures of the components of the two samples were calculated using the Antoine equation, and are presented in Tables S3 and S4. The constants for the Antoine equation were obtained from various resources^47,48^. Finally, the flammability diagram was drawn to determine the temperature at which the vapour mixture would become flammable. Table S5 presents the detailed calculations for the new oil sample. As indicated by this table and Fig. 5, this mixture is flammable at 77 °C. This ignitable (flammable) mixture could be ignited if mixed with air in the presence of a source of ignition such as a static electricity discharge, flame, electrical arc, or similar. The same procedure was followed to determine the temperature at which the used oil sample could produce a flammable mixture; Table S6 presents the detailed calculations. As indicated in this table and in Fig. 6, the mixture would become flammable at 115 °C.

## Conclusions

In electrical substations, accidents are often caused by more than one contributing factor, such as design defects, sudden power surges, winding failures, insulation oil leaks, and the formation of ignitable mixtures. In this study, the risk of fire and explosion accidents in electrical substations was investigated. New and used mineral oil were collected from an electrical substation in Riyadh, and their compositions were determined using GC–MS. The GC–MS analysis revealed that the majority of the components of the two samples were alkanes, alkenes, and alkynes. Using the GC–MS results, the flammability limits of the mixtures were predicted at 25 °C and their flammability diagrams were constructed; the diagrams indicated that the vapour mixtures of the two samples were not flammable at 25 °C. A further investigation was performed to identify the temperature at which each vapour mixture would become flammable. The results revealed that the vapour mixtures of the new and used oil would become flammable at 77 and 115 °C, respectively.

The findings of this study provide a useful assessment of the temperatures at which flammable mixtures will be formed. They could assist in the prediction of fires and explosions in electrical substations.

## Supplementary information

supplementary information.

supplementary information 2.
