# Supplementary material for: Risk of Fire and Explosion in Electrical Substations Due to the Formation of Flammable Mixtures
Source: Sci Rep. 2020 Apr 14;10:6295. doi: 10.1038/s41598-020-63354-4 (PMC7156400; doi:10.1038/s41598-020-63354-4)
Supplement: Supplementary file 2 — supplementary information 2. [file 41598_2020_63354_MOESM2_ESM.docx]

**Risk of Fire and Explosion in Electrical Substations Due to the Formation of Flammable Mixtures**

Mohanad El-Harbawi*, Fahad Al-Mubaddel

Department of Chemical Engineering, King Saud University, Riyadh, 11421, Saudi Arabia

*Email: [melharbawi@ksu.edu.sa](mailto:melharbawi@ksu.edu.sa)
